# The Measurement of Physical Resiliencies: Conceptualization, Study Design, and Early Data

**DOI:** 10.1093/geroni/igaa057.3029

**Published:** 2020-12-16

**Authors:** Karen Bandeen-Roche, Ravi Varadhan, Brian Buta, Jeremy Walston

**Affiliations:** 1 Johns Hopkins Bloomberg School of Public Health, Baltimore, Maryland, United States; 2 Johns Hopkins University, Baltimore, Maryland, United States

## Abstract

This talk presents the conceptual framework, study design, and pilot data for the Study of Physical Resilience and agING (SPRING). SPRING aims to develop signatures of physical resilience in older adults who will undergo clinical stressors; validate the signatures’ ability to distinguish those who will respond resiliently; and characterize underlying physio-biological determinants. The underlying physiology is conceptualized as a dynamical system, and resilience, as a property thereof. The SPRING pilot has assessed n=77 older adults before, during and repeatedly after experiencing knee replacement surgery, incident hemodialysis, or bone marrow transplant; a confirmatory study evaluating n=100 older adults per each of these stressors is underway. Resilience signatures grounded in dynamical data from multiple stress-response assessments will be presented. So also will resilience phenotypes—longitudinal functional trajectories over the study visits: These show considerable heterogeneity within and across stressors. If successful, our study will open the way for interventions to bolster resiliency. Part of a symposium sponsored by Epidemiology of Aging Interest Group.

